# “Chili Burn”—A Case Report of Contact Dermatitis Caused by *Capsicum* Peppers

**DOI:** 10.3390/life15040539

**Published:** 2025-03-26

**Authors:** Maja Hitl, Katarina Radovanović, Nebojša Kladar

**Affiliations:** 1 Department of Pharmacy, Faculty of Medicine, University of Novi Sad, 21000 Novi Sad, Serbia; katarina.radovanovic@mf.uns.ac.rs (K.R.); nebojsa.kladar@mf.uns.ac.rs (N.K.); 2 Center for Medical and Pharmaceutical Investigations and Quality Control, Faculty of Medicine, University of Novi Sad, 21000 Novi Sad, Serbia

**Keywords:** Hunan hand, dermatitis, paprika, capsaicin

## Abstract

Peppers (*Capsicum* spp.) represent not only a plant with a demonstrated history of diverse medicinal applications but also a species having non-neglectable adverse effects potential. “Chili burn” or Hunan hand syndrome represents a type of contact dermatitis rarely appearing after using chili peppers. Here, a case of “chili burn” with no specific treatments or sequelae is presented. A young woman presented with contact dermatitis after first- and second-time dermal exposure to a chili pepper. A strong burning sensation appeared shortly after on the hands and around the mouth after exposure to the plant. The patient applied non-specific measures (hand washing with mild soap and rinsing the affected areas with acidic solutions) with minor improvement; finally, the “chili burn” resolved itself. No other medicines were applied, and no consequences were recorded. Although rare, the use of chili pepper has the potential to cause contact dermatitis. The awareness of medical professionals of this entity should provide adequate diagnosis and treatment for patients.

## 1. Introduction

The herbal family Solanaceae includes many plant species considered important from pharmacological and toxicological points-of-view (e.g., deadly nightshade, jimsonweed, or henbane) and also many plants frequently used in human nutrition, such as potatoes, tomatoes, and aubergines. The genus *Capsicum* also belongs to the Solanaceae family and is represented by various sorts of peppers (sometimes called paprika). Some of the most common are chili peppers, jalapeños, red peppers, and many others, which are famous for their culinary value [1]. The entities often reported as valuable sources of compounds having medicinal applications include the fruit of *Capsicum annuum* L. var. minimum (Miller) Heiser and small-fruited varieties of *Capsicum frutescens* L., which are used for the production of various extracts, and further formulated as pharmaceutical dosage forms [2]. These should not be mistaken for possibly more commonly used spices, such as black or white peppers, as these species belong to the Piper genus and are not related to the Solanaceae family.

The main active ingredients in *Capsicum* peppers are capsaicinoids. These compounds are products of secondary metabolism and are classified as protoalkaloids. They include capsaicin, dihydrocapsaicin, nordihydrocapsaicin, homodihydrocapsaicin, and homocapsaicin, with the first compound being the most recognized and abundant [3]. Capsaicin, its derivatives, and (semi)synthetic analogs can be considered pharmacologically active ingredients, which allow the use of these compounds, as well as *Capsicum* species, as their natural-occurring source in the treatment of several conditions and diseases. Primarily, they find their place in the treatment of pain, including neuropathic pain, musculoskeletal pain, and migraines. Furthermore, capsaicin is used for its anti-cancer activity, anti-obesity effects (via regulating carbohydrate and lipid metabolism and oxidative stress), and beneficial effects recorded in the treatment of some cardiovascular diseases and events (e.g., in myocardial injuries in infarct and affected platelet activity) and neurogenic bladder and painful bladder syndrome. Additionally, studies are being conducted to explore the therapeutic potential in labor pains, Alzheimer’s disease, asthma, rhinopathy, and many others [4,5]. Besides all those previously mentioned, some dermatological conditions (such as pruritus of various etiologies, notalgia paresthetica, alopecia areata, lichen simplex chronicus, psoriasis, etc.) have seen treatment attempts made with topical forms containing capsaicin [4,6].

Regarding the adverse effects, they are recorded for both *Capsicum* herbal drugs and capsaicin. The application of capsaicin (or paprika) has been previously associated with adverse effects appearing on the skin. Besides reported anaphylaxis, rhinitis, conjunctivitis, and asthma, several cases of contact dermatitis (mainly mediated by allergic mechanisms) have been reported [1]. Here, a case of contact dermatitis appearing on hands caused by first- and second-time exposure to chili paprika (“chili burn”) is presented, with no specific treatment being applied.

## 2. Detailed Case Description

**Figure 1:**
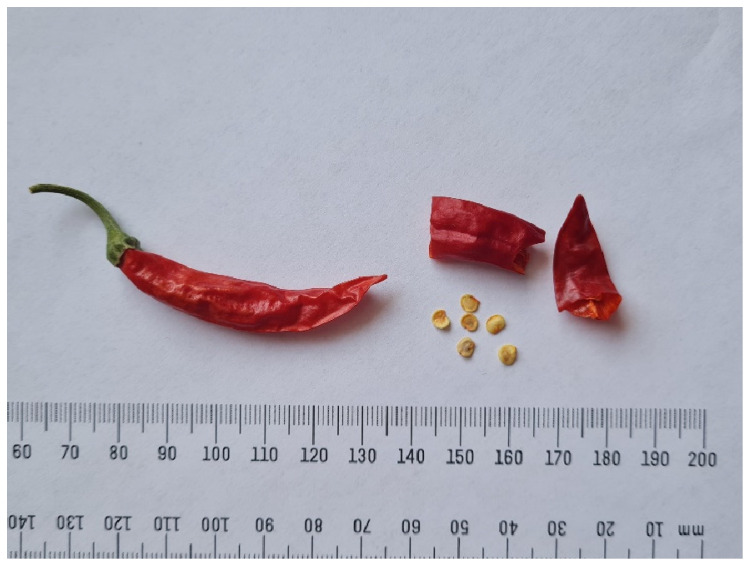
The appearance of the dried chili pepper, as provided by the patient. The picture was taken at the Laboratory for Pharmacognosy and Phytotherapy, Department of Pharmacy, Faculty of Medicine, Novi Sad. The species was identified as *Capsicum annuum* L. 1753 var *annuum*.

A 34-year-old woman presented with hand dermatitis. Although the patient reported eating spicy food previously, she stated that this was her first time chopping home-grown chili peppers and having hand contact with them (the dried pepper is presented in Figure 1).

**Figure 2:**
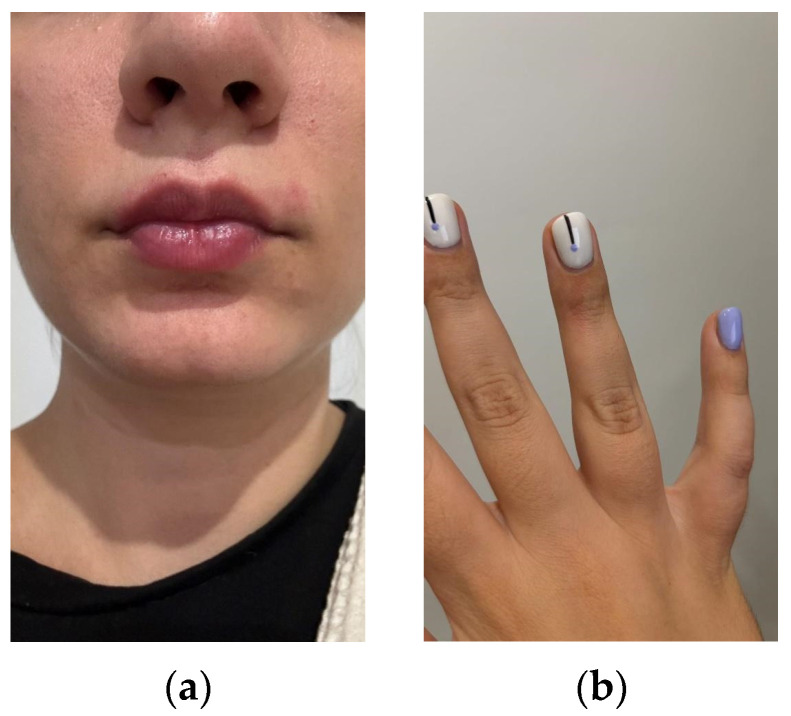
The erythema around the mouth (**a**) and on the finger (**b**). The pictures were taken by the patient at the time of the second exposure.

A couple of hours after preparing the meal, the patient started feeling a strong burning sensation radiating from the fingers up to the entirety of both hands, the sensation being stronger in the right hand, with the patient being right-handed. She reported the highest level of pain in her thumbs and index fingers. The patient described her pain as being 7 on a scale of 1 to 10, with 10 being the strongest pain imaginable. Erythema was not noticed on the hands. No abrasions or other injuries to the skin on the hand were present. To relieve the burning, she repeatedly washed her hands with cold water and mild soap. Moreover, the pain intensified after the patient used head shampoo for hair washing. No other remedies other than cold water were applied, and the burning sensation stopped after several hours. After approximately 10 days, the patient self-opted to repeat the exposure to the chili pepper. The burning sensation appeared in the fingers again, for a shorter period of time and in different parts of the fingers, followed by the pain classified as 6 according to the previously mentioned subjective pain scale. The patient also bit the pepper, and the erythema appeared around the mouth and later on the finger surface (Figure 2).

She treated the areas with cold water, and some relief was obtained, although only for a short period of time. The patient also tried following advice found online, which suggested submerging the fingers in an acidic solution—she used the solution from pickled cucumbers. This action resulted in an increase in pain, and after this, she repeatedly rinsed her hands in cold water. No other medicines were applied. The patient reported to the authors of the paper only after the two events described. Additionally, the woman was asked about her medical history, especially related to dermatological disorders. She denied any allergies or any other skin disorders, except for a total of two episodes of idiopathic rashes after sun exposure, which self-resolved after a short period of time without the application of medications. The patient reported no food, beverage, drug, or medicinal herb allergies. Since the patient reported to the authors of the paper approximately two months after the second recorded event, no dermatological testing was performed, nor was blood analysis performed.

The patient was advised to limit exposure to the chili pepper. She stated that, from now on, she plans to avoid handling the chili entirely and leave it up to other people to dice peppers if necessary for a dish. Finally, the patient was advised to seek medical evaluation by a dermatologist in case of repeated dermatitis appearance.

## 3. Discussion

The spicy chili taste is something that is frequently desired in various dishes and cuisines. This is often studied in gastronomy and psychology due to the extremes sometimes present among consumers [6,7]. Often misinterpreted as taste, thus suggesting interaction with taste buds which are located on the tongue and are responsible for the taste of sweet, salty, acidic, bitter, and umami [8], the sensation of chili or spice is mediated via pain receptors. Capsaicin found in peppers bonds to the transient receptor potential vanilloid 1 (TRPV1) and triggers impulses via polymodal C and Aδ nociceptive fibers [3,4], which results in pain and heat sensations. At the same time, the chili taste can be seen as a desired and as an adverse effect. The effect caused by *Capsicum* peppers is recognized among the general population, and unfortunately, there are also recorded cases of *Capsicum* pepper abuse, precisely for their burning sensations. Specifically, a case report of parents forcing their underage children to hold split jalapeño peppers in their mouths is recorded. This resulted in intense pain in their mouth, throat, stomach, anus, and other gastrointestinal discomforts [9]. Another, more widely known (ab)use of pepper is in the form of pepper spray, a product which can be used by an individual for attack or self-defense purposes, but also as a “riot control incapacitating agent,” i.e., in political and other types of public protests and events. These formulations contain oleoresin, a liposoluble product obtained from the plant [10]. The majority of injuries caused in such a manner are classified as minor or moderate, although the potential for extensive wounds is documented [11].

A search for previously reported cases of “chili burns” was performed. Databases and online repositories PubMed, Science Direct, Google Scholar, and ResearchGate were searched using the terms “chili”, “Hunan hand”, “*Capsicum*”, “pepper”, “burn”, “dermatitis”, “case report” and their logical combinations. Accessible articles written in English were evaluated, and their references were also inspected for potential additional articles. No restrictions regarding the date of publication or the gender, age, or race of the patients were applied. The retrieved case reports, in which dermatological entities were classified by the attending physician as dermatitis, are presented in Table 1.

Two of the presented case reports from Table 1 highlight the appearance of adverse effects related to professional exposure. Namely, frequent exposure to chili resulted in “chili burn” being described as “deep, aching, shooting pains” [14]. Another case of occupational allergic contact dermatitis was recorded in a patient who was chronically exposed to a mixture of spices containing paprika. Erythema, edema, and scaling first appeared on the hands, further spreading to several segments of the face, and dermatological tests confirmed *Capsicum* as the causative agent [15]. Moreover, testing performed in two reported cases of contact urticaria and anaphylaxis, suspected to be caused by the *Capsicum* species (not presented in Table 1, as they were classified as other than dermatitis by the attending doctor) [17,18], confirmed *Capsicum* as the allergen. Regarding the previously reported cases of “chili burn” (presented in Table 1), the application of a patch test in only one case enabled the classification of contact dermatitis as allergic. Still, it is well known that herbs and their extracts contain diverse classes of compounds, among which many have the potential to act as allergens. However, the lack of dermatological testing leaves the possibility that contact dermatitis caused by *Capsicum* is an irritant. Moreover, since capsaicin is a highly selective agonist, it can be hypothesized that precisely TRPV1 activation may result in irritation, pain, and inflammation [4].

The first case of the previously described *Capsicum* dermatitis dates back to 1981 when a patient presented redness and pain in the hands after preparing a Chinese cuisine dish containing red pepper. Presumably, this event triggered the naming of the described clinical condition as Hunan hand syndrome [12]. Shortly after, a report on how to treat “chili burn” advised immersing the affected area in vinegar (5% acetic acid) no longer than 30 min after exposure [19]. This advice seems to contrast with the currently presented case, as exposure to the pickled solution worsened the burning sensation. Bearing in mind that capsaicin belongs to alkaloids, compounds with mild alkaline properties, it could be expected that the application of acidic solutions (e.g., vinegar, pickled solution, lemon juice) results in the neutralization of capsaicin (and its effect). However, patients’ experiences in studied cases seem to be divergent since some report alkaline compounds (such as baking soda paste) to reduce capsaicin-induced pain. Other therapeutic approaches are also reported. A case report of a patient with “chili burn” stated that immersion in cold water did not help in alleviating the symptoms; the patient was advised to apply an alkaline solution (baking soda paste), rubbing alcohol, and corticosteroid ointment, yet no satisfactory result was obtained; the patient waited until the pain resolved by itself [13]. Another patient was given similar advice—cold water, baking soda paste, and corticosteroid ointment—yet no relief was obtained until the patient was treated with continuous stellate ganglion block and gabapentin [14]. The latest reported case of Hunan hand syndrome was treated similarly. Since the application of peroral non-steroid anti-inflammatory drugs and topical corticosteroids, as well as subsequently applied morphine, had no effect, and the combination of morphine and ketamine displayed a mild sedation effect, the treating doctor prescribed pregabalin, which was successful in reducing the pain [16]. Interestingly, gabapentin and pregabalin are two drugs whose application resulted in the relief of symptoms for patients in two previously reported cases [14,16] where other drugs were less or completely ineffective. Gabapentin and pregabalin, drugs used in cases of neuropathic pain, affect the entry of calcium in neurons, subsequently leading to reduced release of neurotransmitters and, finally, the reduced sensation of pain. As capsaicin bonds to TRPV1 and causes the release of neurotransmitters (such as substance P and calcitonin gene-related peptide), it is understandable why drugs antagonizing this effect are adequate choices for pharmacological treatment [14,16].

A common piece of advice after the adverse effects of chili peppers, which is more frequently found in populistic literature and on the web, is the use of milk, applied both for drinking and on the skin. This is possibly because milk represents an emulsion containing fatty acids and triglycerides, which could aid in co-dissolving the hydrophobic part of the capsaicin molecule, as well as other capsaicinoids [3,20]. However, a recent study found that milk’s fat may not be the only compound contributing to this effect. After testing several foods and water, it was demonstrated that temperature, as well as fat, sugar, and protein content, all contribute to the reduction in oral burn induced by capsaicin [21].

**Figure 3:**
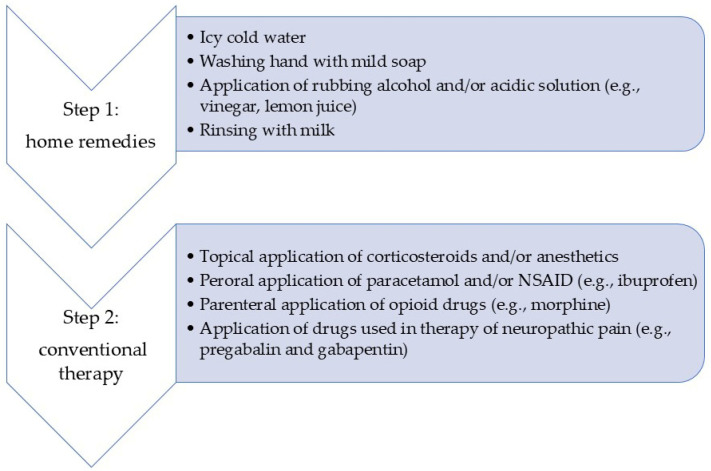
The suggested “chili burns” treatment is based on reported therapeutic approaches, as given in reported cases. NSAID refers to non-steroidal anti-inflammatory drugs.

Final considerations, taking into account the presented case reports, suggest no uniform treatment approach for “chili burns”, but only the existence of a two-step gradual therapy consisting of household items, and in the case of lack of efficacy, further proceeding to conventional drugs. Therefore, the suggested management of “chili burns” is given in Figure 3.

In the presented case, the examination of the patient by a corresponding health specialist and dermatological tests confirming/excluding the mechanism of dermatitis are lacking; the patient was advised to reach out to a doctor in case of repeated exposure to chili and the appearance of dermatitis, to obtain adequate medical care in case she needs it. So, why should an emergency doctor or dermatologist be aware of the “chili burn”? While medicinal plants can successfully be used to prevent and treat contact dermatitis [22], there are two sides to this coin—medicinal plants can also be the causative agents of contact dermatitis [23]. Moreover, additional problems are created when a plant with medicinal potential is regularly used in everyday life and nutrition. *Capsicum* species are frequently used by the general population and are highly abundant in several cuisines, which emphasizes the importance of knowledge regarding the potential of pepper to cause adverse effects on the skin. The existence of such awareness among medical professionals is mandatory for providing adequate care to patients in need.

## 4. Conclusions

Handling *Capsicum* peppers may result in contact dermatitis, although rarely. This adverse effect may sometimes require special medical intervention and usually does not leave long-term consequences. However, medical professionals should be aware of the plant’s potential to cause “chili burn” in order to give adequate medical advice regarding potential treatment and caution to be taken in the future when peppers are considered for both medical and dietary purposes.

## Figures and Tables

**Table 1:** Previously reported cases of dermatitis caused by *Capsicum* peppers.

Reference	Age (Years) and Sex of Patients	Clinical Evaluation	Attempted Self-Treatment	Treatment	Additional Comments
Weinberg, 1981 [12]	32, M	tachycardia (>120/min) intensive perspiration intensive agitation	ice water immersion	lidocaine gel applied to fingertips	patient reported skin abrasion due to the use of sandpaper for furniture
Williams et al., 1995 [13]	31, F	highly distressed physiological heart rate, body temperature, and respiration	ice water immersion washing hands	repeated ice water immersion, rubbing alcohol, baking soda paste, fluocinonide ointment	patient reported an allergy to penicillin patient reported no improvement after the use of the suggested treatment until the pain resolved itself
Saxena and Mandhyan, 2013 [14]	42, F	physiological heart rate, blood pressure, body temperature, and respiration	ice water immersion washing hands	repeated ice water immersion, cooling gel pack, baking soda paste, fluocinonide ointment continuous stellate ganglion block and gabapentin	dermatitis appeared as a result of professional exposure patient reported an allergy to sulfa-drugs
Lambrecht and Goossens, 2015 [15]	23, M	patch testing to spice mixture containing paprika and diluted capsaicin	none	none	dermatitis presented on the hands and several segments of the head dermatitis appeared as a result of professional exposure; after the change of workplace, no further dermatitis was recorded
Perrier et al., 2024 [16]	31, F	tachycardia	ice water immersion milk immersion flour immersion	peroral paracetamol, ketoprofen and nefopam, topical silver sulfadiazine morphine, morphine + ketamine pregabalin	patient reported re-appearance of symptoms at the thought or sight of peppers (suggestive of post-traumatic stress disorder)

M—male, F—female.

## Data Availability

The original contributions presented in this study are included in the article. Further inquiries can be directed to the corresponding author.
